# Infrared Thermography Images Acquisition for a Technical Perspective in Screening and Diagnostic Processes: Protocol Standardized Acquisition

**DOI:** 10.7759/cureus.19931

**Published:** 2021-11-27

**Authors:** Stefano Politi, Anna Aloisi, Vittorio Bartoli, Antonio Guglietta, Fabrizio Magnifica

**Affiliations:** 1 Aerospace Medicine, Diagnostic Therapeutic and Rehabilitative Aeromedical Center, Italian Air Force, Rome, ITA; 2 Physiotherapy, Motion Rehab, Itri, ITA; 3 Neurology, Sapienza University, Rome, ITA

**Keywords:** thermography, sars-cov-2, temperature, thermal imaging camera, physiotherapy

## Abstract

In this technical report we describe the thermographic setting protocol suitable for the FLIR T650SC thermal imager (FLIR Systems, Inc., Wilsonville, OR), an instrument that detects electromagnetic radiation in the infrared field which is physiologically emitted from the human body. FLIR T650SC thermal imager processes infrared radiations graphically and analyzes them through a specific software. In biomedicine, infrared thermography is a promising technique amongst other conventional methods used for detecting skin temperature differences considered as a possible sign of disturbances in the human body. Currently, automatic screening of temperature from a safe distance is an instrument utilized in the front line of the SARS CoV2 emergency.

The processing method of the thermogram considers an initial setting of constant parameters that cannot be subsequently modified such as temperature range, focusing and image composition. After the acquisition variable values important in the processing and analysis of the thermogram, such as detection of environment temperature, reflected temperature, emissivity, relative humidity and contrast palette, are set in the software. The analysis is performed using the FLIR Tools software.

In the biomedical field standardized acquisition of thermograms facilitates the identification of trigger points and areas of hyper- and hypothermia distributed on the skin surface and muscle bundles. The protocol made it possible to create images with the same acquisition method for all patients.

The thermal imaging camera is a valid screening tool because its execution is rapid, it is non-invasive, well-tolerated, and at a low cost for patients.

## Introduction

Temperature is a well-established health indicator [1] and humans, homeotherms, are capable of living maintaining a constant temperature different from that of the surrounding environment. This phenomenon, vital for the conservation of a regulated internal environment, is known as homeostasis and involves the functions and the composition of fluids and tissues. Variations in the internal temperature of more than a few degrees, in terms of increase or decrease, are a clear indicator of a homeostatic dysfunction sufficient enough to put at risk the chemical processes essential in the body [2]. Body temperature can be measured locally by a mercury thermometer or by thermocouple placed in physical contact, or through an infrared camera which detects the natural thermal radiation from the body and is capable of producing an image that represents the distribution of temperature areas of the body. Infrared radiation, identified by wavelengths between 0.75 and 14 μm [3], lies between the portion of the visible electromagnetic spectrum and that of the microwaves. The main source of infrared radiation is heat or thermal radiation. Any object or subject, such as the human body, at a temperature above absolute zero (-273.15°C or 0 K), emits radiation in the infrared area. Even objects considered to be very cold, such as ice cubes, emit infrared radiation [4]. Since William Herschel discovered infrared radiation and recorded the first thermal image of his son [5], thermography has gained diagnostic importance for the human body and the term “infrared thermography” (IRT) was used for the first time especially in medical sciences [3]. The potential of infrared radiation can also be applied in screening activity. It has been demonstrated by its current use in the actual SARS-CoV-2 emergency in which the measurement of the body temperature at a safe distance constitutes one of the first containment measures and protection for both healthcare professionals and patients. The cameras of a radiation infrared measure the thermal radiation emitted by any surface and convert them into thermal images. In the resulting thermograms, it is possible to highlight the temperature differences with contrast palettes available in the camera software. The energy emitted in the infrared spectrum is focused by the optical fibers towards a detector; this detector sends the information to the electronic sensor for image processing and then the software translates the data from the detector into an image, called thermogram, observable directly by the operator in the thermal imager display and, simultaneously, onto a screen. FLIR thermal imaging models are sensitive enough to detect changes in temperature below 0.02°C (273.17 K), allowing precise and quantitative measurements reliable for the detection of dysfunctions affecting the organism [6]. The third generation of infrared cameras currently available uses the Focal Plane Array (FPA) device associated with thermal, cooled and uncooled, detectors of photons [7].

A recent study shows how IRT has expanded its uses by demonstrating to be effective in many biomedical and sports fields [6]. In a study by Oliveira et al. [8], the diagnosis and classification of injured ankles have been carried out using IRT. Evaluation of the benefit of infrared thermography as a diagnostic tool for injury’s degrees was the main focus of the study presented. The uncooled infrared camera FLIR E60 SC (FLIR Systems, Inc., Wilsonville, OR), with certain specifications, was used to acquire the thermograms. FLIR camera’s specifications are FPA of 320×240, NETD (thermal sensitivity) of 0.05°C at 30°C, accuracy of ±2% of the general temperature reading, long wavelength (7-13.5 μm) and using a field of view (FOV) minimum focus distance 25° x 19° / 0.4 m (1.34 ft). Consequently, a high potential for validating thermography IR for the diagnosis of ankle injury has made an important contribution to the literature such as classification indicator. Chudecka and Lubkowska [9] used thermal imaging instruments to reveal the thermal maps of young men and women for medicine, physiotherapy and sports. The authors have established temperature ranges and distribution on the body surfaces of men and women. Every thermogram of each participant was recorded by the infrared camera model ThermaCAM SC500 (FLIR Systems, Inc., Wilsonville, OR) in an upright position. They performed measurements in the afternoon after 4 pm. In this study, the important parameters were BMI, body surface PBF and body mass. Araujo et al. [10] studied the symbolic data analysis framework (SDA) to evaluate breast abnormalities, a major disease in women, detecting simultaneously breast cancer. In order to classify breast abnormalities, the authors proposed three gradual processes which are the acquisition, segmentation and morphological elaboration of thermographic images. In the image acquisition phase, all the thermograms of a group of patients were captured by the FLIR S45 infrared camera (FLIR Systems, Inc., Wilsonville, OR) in a hospital in Brazil. The authors transformed each thermal image into a temperature matrix to extract breast thermogram areas concluding that thermography is one of the complementary methods for breast screening before mammography evaluation. In a study by Anzengruber et al. [11], the primary objective was to evaluate a new method of non-contact infrared reading of patch tests, and the secondary objective was to identify a possible correlation between the intensity of the patch test reaction and the change of skin temperature. The authors highlighted that the use of IR screening has proven to be reliable in distinguishing between an allergic and an irritant reaction in the diagnosis of contact dermatitis (ACD), given that the difference in average temperature between the two groups was found to be highly significant (p < 0.0001). By administering the patch test for allergen detection, the IR image showed a clear difference between an irritating reaction, with an increase in the local skin temperature of only 17 ± 0.31 C with respect to the surrounding one, and an allergic reaction, with a more significant increase in the skin temperature that has settled around 0.72 ± 0.67 C relative to the surrounding one. Paterson et al. in 1978 and subsequently Akerman and Kopp in 1988 analyzed, using thermograms, the thermal signs of rheumatoid arthritis on different joint structures. The results obtained in the thermographic analysis showed that the measurement was influenced by the quantity of bone tissue present in the analyzed area (for example length and thickness of the bone) since it changes the coefficient. These measurements, therefore, require the calculation of the percentage of heat retained by each type of bone, which must be converted into a correction coefficient for the evaluation of the inflammatory pathology in the different joint districts. From these results, from Paterson et al. and Akerman and Kopp, it emerges that, while the connective tissues have a homogeneous composition in the different districts, the analysis of the bones provides a sensitive measurement due to the intrinsic diversity of this tissue with respect to heat emission [12,13].

The aim of this paper is a technical report describing the setting procedure for the FLIR T650SC thermal imaging camera (FLIR Systems, Inc., Wilsonville, OR) on patients complaining algic symptoms in the vertebral spine, in order to provide standard acquisition and processing parameters, to guarantee the maximum reliability of the measurements obtained by this instrument already used in the evaluation and in the rehabilitation process of patients with spinal disorders [14-16]. The object of this standardization is to obtain reliable thermograms in biomedical screening. The data acquisitions and their processing were conducted in the Therapy Section and Rehabilitation of the Aerospace Medicine Department of the Italian Air Force (Rome), where every subject was made aware, through the administration of an informed consent form, of the characteristics of the analysis by IRT. The data was collected from patients whose symptoms were located in the cervical region, often associated with limited cervical movement associated with an arthritic degeneration. This inflammation may be the result of postural changes with an imbalance of the muscles of the cervical spine often caused by a prolonged incorrect postural attitude.

## Technical report

**Figure 1:**
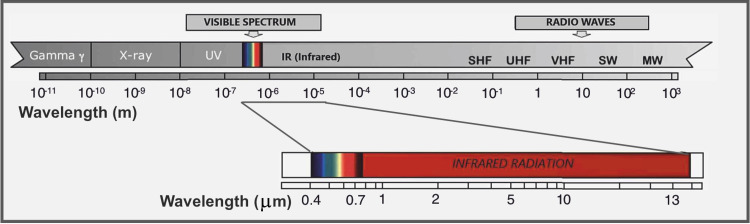
The image represents the positions of the infrared radiation in the spectrum of electromagnetic waves. SHF: Super high-frequency radio waves; UHF: Ultra high-frequency radio waves; VHF: Very high-frequency radio waves; SW: Short waves; MW: Medium waves.

This study was conducted in accordance with the Declaration of Helsinki. In this report, we describe the procedure for the acquisition of standardized thermograms with the FLIR T650SC camera, defining the load settings of the constant parameters and variable values required for IRT acquisition [17]. As human eyes, the thermal camera is sensitive to electromagnetic waves. The difference is that the thermal camera detects the heat that the human eye cannot see, but can perceive with other senses. Indeed, human eyes see the wavelength of the visible, between 0.4 and 0.7 while a thermal imaging camera is sensitive to infrared radiation (Figure 1) [4]. The standardized thermogram acquisition phase is preceded by the preparation and positioning of the subject and the machinery itself. The preparation of the subject includes the removal of all the clothes except for Lycra shorts and of any object on the body surface, and finally, acclimatization.

Acclimatization is a very important step to allow the subject to stabilize the body temperature and adapt it to the surrounding environment. The achievement of thermal equilibrium is aimed at avoiding any alteration of the thermographic analysis due to the influences of the patient's home environment. In the literature different periods of equilibrium are defined ranging from 10, 20 or even 60 seconds. In this report, a subject acclimatization period of 20 seconds was adopted. At the end of acclimatization, the patient is placed in front of a wall with a non-reflective surface [17]. For the analysis of the spine, it is necessary to expose the entire dorsal surface of the trunk which must be facing the thermal imager. The subject must maintain an upright position with the head facing forward and the arms positioned at the sides at rest. This position must be reached without being touched by the operator and asking him not to touch himself to avoid prolonging acclimatization. In fact, the thermal trace released by the patient's hand or by that of the operator, takes further time to not be detectable by the thermal imager. At this step, the thermal imager is positioned one meter away from the subject and a 90° angle must be maintained between the lens and the body surface to be analyzed. This setting allows an accurate detection that reduces the risk of information loss [18]. After the preparatory phase of the subject and his positioning, “focus”, “image composition” and “detectable temperature range”, set in a range between 26°C and 40°C, are the three non-variable parameters set before acquisition. During the acquisition, images are monitored in real-time to verify the proper focus and composition. When acquisition finished, the phase of frame processing through the software provided started. In the software values of emissivity, room temperature, reflected room temperature, relative humidity and distance from the object must be entered.

**Figure 2:**
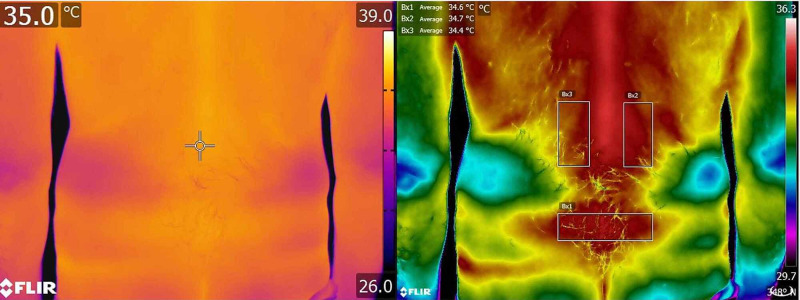
Representative image of the acquisition with the thermal imaging camera. At this level, the operator checks the focus and composition of the image; (on the right) processing of the left image in terms of thermogram. Note that the contrast palette associates a color with a temperature.

The study by Fernàndez-Cuevas et al. in 2015 estimated that the emissivity value (ε) of human skin ranges between ε = 0.97 and ε = 0.99 at wavelengths between 2 and 14 μm. Therefore, ε = 0.98 is the standard value of human skin emissivity. In the acquisitions used for this Technical Report, emissivity value of 0.98 (ε = 0.98) was used. To prevent environmental conditions affecting the validity of the measurements, the conditions of the detection environment were stabilized to prevent sweating and chills in the subject assessed with IRT. The literature reports that the ideal environmental conditions provide for a relative humidity between 40% and 70% and a temperature of 20-22°C [18] and more recent studies such as that of Fernàndez-Cuevas et al. in 2015 recommend positioning the thermal imager about 1 meter from the subject [18]. In this Technical Report, the acquisitions of IRT always occurred in the same environment, at a temperature of 20-22°C and with a relative humidity of 50%. Any influence of direct sunlight on the body and external drafts was carefully avoided. The acquired thermogram was shown on the computer display and processed with the “Iron” contrast palette (Figure 2), set by default in the software. Image analysis and processing were executed with the tools of the pre-installed software.

**Figure 3:**
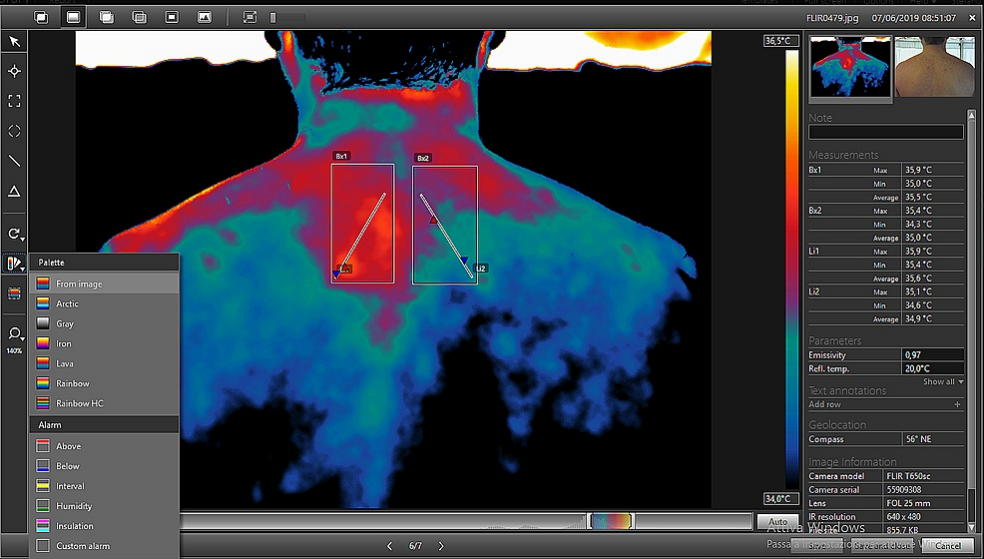
Representative image of a patient of the thermal range bar where thermal tuning is performed, accessible on the FLIR Tools processing program (Bottom right). Menu for selecting the contrast palettes (left).

The purpose of the processing is to identify areas characterized by hypo- or hyper-thermia indices. Also, a thermal tuning could be applied, following the previous settings, by intervening on the span. This operation, performed respecting the temperature range set before the acquisition (26-40°C), aimed to exclude or highlight the areas within a thermal range of interest (Figure 2) [17]. By performing the thermal tuning, both the coldest points and those with elevated thermal values are enhanced. In fact, a greater contrast is created on the surface analyzed in the thermogram to highlight and distinguish the hottest points from the coldest ones, even in the presence of a very low thermal gap below 1°C. After setting the parameters necessary for the correct image processing, images could be displayed with different contrast palette (Figure 3), available in different options depending on the color range. These palettes are essentially of two types: low-contrast or high-contrast. The first is more suitable in areas of the body where there are wide thermal differences, for which it is not necessary to further highlight the thermal information. In this case, the palette chosen must be that with the ideal range of color to highlight the contrast. The second type of palette is used in those areas where the temperature difference is contained in a very small thermal range (Figure 3) [17].

**Figure 4:**
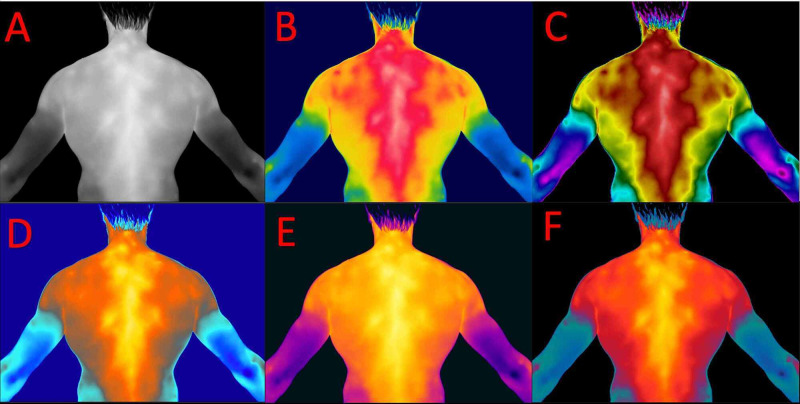
Thermogram image acquired from a patient (A) and processed with the color palettes available on the FLIR Tools software (B-F). A: Gray; B: Rainbow; C: Rainbow* *HC; D: Arctic; E: Iron; F: Lava.

In the case of the human body, the choice of palette varies according to the needs of the operator. The human body surface is extremely homogeneous and therefore the slightest thermal differences must be enhanced through a palette with a wide range of colors in order to obtain an optimal visualization of the thermal gradient that enhances the minimal variations in temperature between small segments of skin that follow one another (Figure 4).

**Figure 5:**
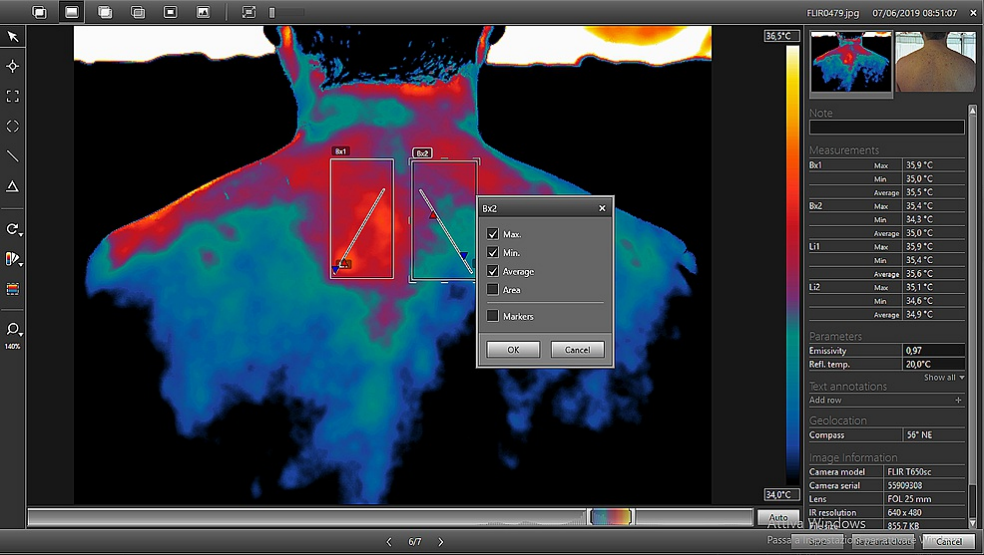
Representative image acquired from the same patient of Figure 4, processed using the Area Measurement Tool (tool box). The menu in the center of the figure allows the selection/deselection of the information that the operator wants to obtain from the area delimited by the box. Indicated with the red circle: command for activating the tool for delimiting areas of interest.

When the thermogram is processed with the choice of the most suitable palette and with thermal tuning by acting on the span and on the temperature range, the analysis of non-homogeneous areas, therefore altered, can be performed using the “Box” tool (Figure 5), that allows to delimit areas of interest.

**Figure 6:**
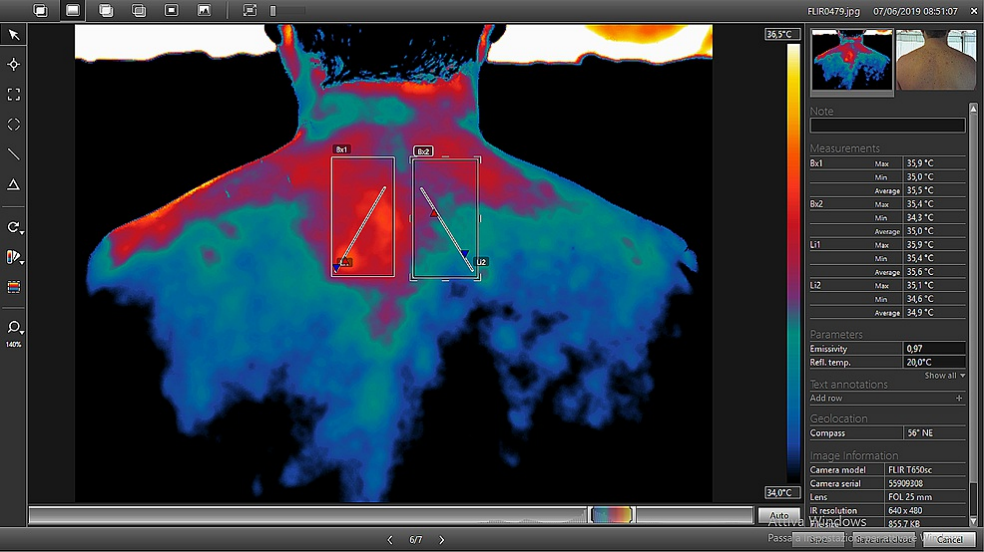
Representative image of the use of the tool box for the comparative temperature measurement of two areas (Bx1 and Bx2) and two profiles defined in each area (Li1 and Li2). Right panel: Measurement table which contains the temperature values measured in Bx1 and Bx2, and along Li1 and Li2. (Max: maximum temperature; Min: minimum temperature; Average: average temperature).

The “Box” tool allows to make squares (selectable by a command on the vertical left toolbar) (Figure 5) of variable width and shape depending on the images obtained. The software provides information about the selected area, such as the minimum, maximum and average temperature [17]. However, by pressing the right button on the box area, information available from the Box is editable selecting or deselecting the different parameters according to the needs. In the evaluation of the spine, a box was inserted in each half of the body in order to have an immediate comparison and to evaluate the thermal gap (Figure 6).

**Figure 7:**
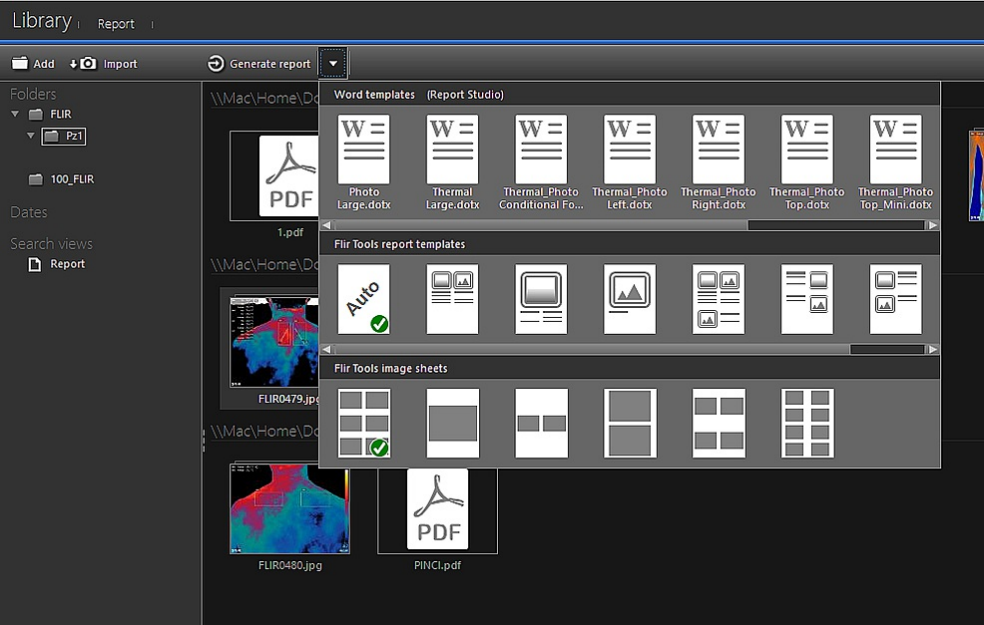
Software screen related to the generation of the thermographic report. Library command allows access to the gallery of images acquired by the thermal imager. From here you can select the image you want to associate with the report; Generate Report command allows the generation of a preset format of the technical report. The arrow next to it allows the choice of different report models and image sheets.

The software allows the use of a “profile” (Li) (Figure 6), that is a straight line that provides information concerning the thermal distribution on each millimeter of the surface on which “Li” extends. This tool can be used alone or together with the Box function (Bx), by entering profiles simultaneously within the same or different areas. Even in this tool, it is possible to select or deselect the parameters concerned. Both for the area measurement tool (Figure 6 indicated as Bx) and for the measurement of the line (Figure 6 indicated as Li) it is possible to display, on the right part of the software screen, a Measurement Table (Figure 6) containing the values ​​of the analyzed areas. Once you have finished processing and analyzing the thermogram, you can save your work and proceed with the creation of the technical thermographic report. By accessing the “Library” section (Figure 7) of the program, you get an overview of all the thermograms acquired with the thermal camera. After selecting the processed image, the creation of the report proceeds.

**Figure 8:**
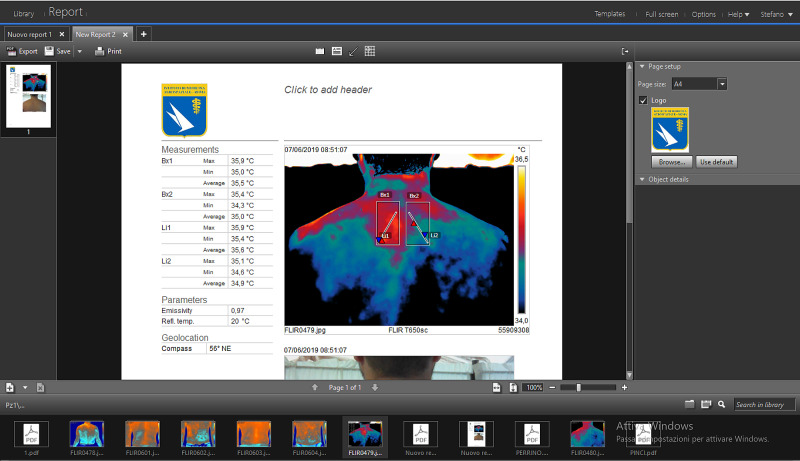
Summary screen of the generated technical report, which automatically displays the table of temperature measurements obtained during the analysis of the thermogram. It is possible to change the page format and insert a logo (top right), add information about the operator (text box, center), enrich the report with other images (bottom).

A toolbar located at the top left contains the “Generate Report” command (Figure 7). In this phase, choosing the command directly automatically processes the report and a default layout (“Auto”) from the software. Another option is to select a drop-down menu (Figure 7) on the right of the “Generate Report” command and choose between the different models present. The software generates a report summary screen of the report (Figure 8). On this work page, the program offers several options: the report contains the Measurements table, the thermogram and the “non-thermographic” image. The software allows to change the arrangement and size of these three elements. In addition, the area measuring instruments (Bx) and the profile (Li) are further editable also within the report sheet. By changing the position and/or the extension of these instruments the Measurements table automatically updates the values.

**Figure 9:**
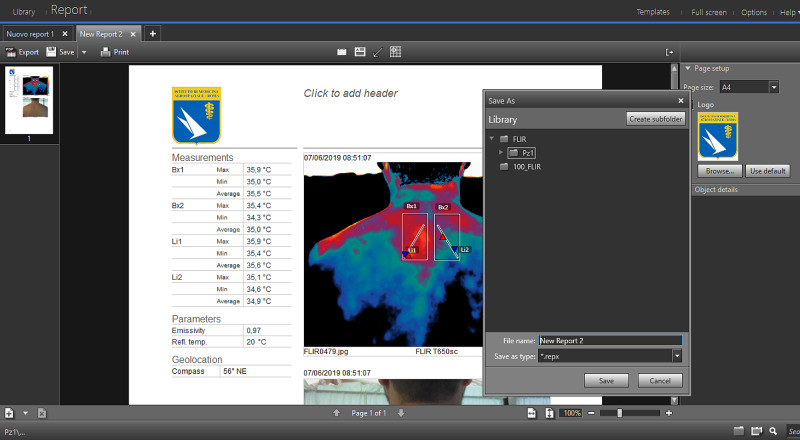
Screenshot of the program containing the “Export”, “Save” and “Print” commands. The menu allows you to change the name of the report and choose its storage destination on the computer.

In the Report, you can place a header using a text box (Figure 8) in which insert any type of content (e.g., patient information, thermographic operator, company/body, etc.). In the format at the top right, also the format of the page can be chosen, between “A4”, “Letter” and “Legal”, while selecting the “Browse” command (Figure 8), a logo (of your company, organization, etc.) can be inserted. This option can deselect by removing the check in the box. At the bottom, you can see a strip containing the image gallery (Figure 8) saved in the software memory. By this tool, another image can be selected and added to the Report. The software automatically inserts the table Measurements, the Thermogram and the “non-thermographic” image of the selected file. Once completed any changes, the report can be saved on the program, exported and/or printed, using the options in the toolbar at the top left. By selecting the “Export” command (Figure 9), the Software saves the Report in PDF format directly in the folder of the patient present on the program and simultaneously opens a PDF file with the Report processed and saved on the PC.

## Discussion

Man is a homeothermic living being, able to maintain a stable internal temperature around 37° ± 0.5 C° regardless of changes in ambient temperature outside where he finds himself. From the point of view of the thermal balance, however, the human body is divided into a central core (“core”) and in a casing or peripheral shell. The central core includes the deep organs of the head, neck, thorax and abdomen, while the outer shell is mainly made up of the skin. The central core manages to maintain a more constant temperature than the outer shell, which is subject to a greater variation in basal temperature, especially in relation to surrounding environmental conditions. Rectal temperature is the best central temperature index. In resting conditions, its value is 37.0°C with an excursion range of ±0.5°C. The swing value depends on gender, age and other factors. In the same subject, in the course of the day, the temperature varies according to a circadian rhythm [18]. The use of the thermal imaging camera is a useful tool in the biomedical field. The information on the thermal state of the tissues, added to those obtained from various analyses and the classic clinical and diagnostic evaluation of the patient, allows a targeted identification and precision of trigger points. Some fields of application of thermography on biological tissues can be, for example, the evaluation of the micro- and macro-circulation, the inflammatory existence or not as a result of joint and muscle trauma, and the state of inflammatory pathologies and degenerative osteoarticular diseases. It also constitutes a non-invasive screening tool that does not expose the patient to any type of radiation and provides a complete and specific picture on which is the surface thermal distribution of the human body. A study by Marins et al. [19] suggests different levels of attention depending on the temperature gap between two symmetrical parts. A difference of less than 0.4°C is to be considered physiological, since small variations are negligible for the organism or could be random errors originated from microclimates present in the surrounding environment or systematic errors caused by the measuring instrument itself. The thermographic evaluation can help the specialist in choosing a path aimed at tissue condition of the patient. Like any instrumental exam, the acquisition and analysis of thermograms must be standardized and reproducible: fundamental parameters for research, diagnostics and follow-up application. Furthermore, recent applications of thermography have taken place to identify the increase in body temperature in the current SARS-CoV-2 emergency, allowing a fast measure and without contact. Their easy use, the real-time vision of the thermographic images, and the guarantee of a measuring range much wider than the IR thermometer, made them instrumentation of primary importance in the identification of subjects in a feverish state, potentially an indicative symptom of being infected from SARS-CoV-2.

## Conclusions

In this technical report, we describe a standardized protocol for technical setting aimed at the acquisition and processing of thermograms using the FLIR T650SC Thermal Camera on the human tissue and aimed at highlighting the thermally non-homogeneous areas with respect to the neighboring ones. This standardization allows reliable thermograms to be obtained as part of the biomedical screening. In addition to the advantage of sensitivity, the thermographic technique allows rapid administration to the patient associated with low costs and non-invasiveness. Furthermore, it is possible to obtain answers and evaluations in a very short time and carry out thermographic follow-ups from a distance and without damaging the patient's health.
